# Drift-Aware Digital-Twin Readiness for Two-Stage Reverse Osmosis: Plant-Scale Time-Aware Machine Learning and Operating-Regime Validation

**DOI:** 10.3390/membranes16100328

**Published:** 2026-09-30

**Authors:** Smail Es Sellami, Abdeslam Taleb, Mohammed El Hachoumi, Abdelkader Es Sellami

**Affiliations:** 1 Laboratory of Process Engineering and Environment (LGPE), Faculty of Sciences and Techniques of Mohammedia (FSTM), Hassan II University of Casablanca, Mohammedia 28806, Morocco; abdeslam.taleb@etu.univh2c.ma (A.T.); mohammed.elhachoumi-etu@etu.univh2c.ma (M.E.H.); 2 JESA S.A., Casablanca 20270, Morocco; abdelkader.essellami@um6p.ma

**Keywords:** reverse osmosis, membrane process, digital twin, machine learning, operating-regime drift, time-aware validation, predictive monitoring, data quality

## Abstract

Reverse osmosis (RO) digital twins require predictive models whose accuracy remains credible as plant operating conditions change. We evaluated the readiness of predictive models for a full-scale, two-stage RO plant using operational spreadsheets and temporally structured validation. An audit of 70 legacy worksheets identified heterogeneous layouts. A normalized source provided 476 train-day records from 17 operating months between June 2022 and November 2023, while 55 records from April and May 2024 formed an independent later-period holdout. Four supervised regression algorithms (Ridge Regression, Random Forest, Extra Trees, and Gradient Boosting) were evaluated for second-stage permeate flow and conductivity. Under a random split, Random Forest achieved R^2^ values of 0.876 and 0.833, with root mean square errors of 8.48 m^3^/h and 63.26 µS/cm, respectively. Chronological validation reduced R^2^ to 0.042 and 0.118. On the later holdout, R^2^ fell to −1.173 and −0.918, with corresponding root mean square errors of 11.14 m^3^/h and 218.65 µS/cm. Rolling-origin tests revealed substantial variability among successive periods. Distributional diagnostics identified changes in feed pressure, first-stage permeate conductivity, second-stage flow, and second-stage conductivity; the available data could not establish their physical causes. These findings show that performance under random splitting is insufficient to establish readiness for future use. A membrane-process digital twin requires traceable data, chronological evaluation, operating-regime surveillance, controlled model updates, and human-supervised advisory operation.

## 1. Introduction

Reverse osmosis (RO) is widely used to produce drinking water from brackish and saline sources. Its performance depends on feed composition, driving pressure, temperature, recovery, pretreatment, fouling, scaling, and membrane condition [1,2,3]. A recent fouling-modelling review also documents the differences between mechanistic assumptions and data-driven approaches [4]. Full-scale RO conditions change with time, while process measurements and intervention records may remain scattered across spreadsheets, laboratory reports, maintenance notes, and control systems.

A digital twin connects a physical asset with a maintained digital representation and information exchange [5,6]. Model uncertainty, integration, and maintained predictive validity require explicit treatment [7]. Water-sector applications range from wastewater and ultrafiltration twins to monitoring and data platforms [8,9,10,11,12,13]. For drinking-water systems, Janjua and colleagues described nonstationarity and the importance of updating predictions after deployment [14], whereas Richards and colleagues emphasized governance and oversight when deploying water-sector AI [15]. These experiences motivate an RO-specific assessment of prediction reliability beyond the historical fitting period.

Machine-learning applications in water treatment and desalination have been surveyed previously [16,17,18]. Plant-based RO studies have explored membrane performance and salt rejection [19], dynamic process identification from historical measurements [20], and the relevance of process variables and Random Forest methods for membrane performance [21]. A seawater RO study developed conductivity models using physicochemical and operating variables [22]. Energy-performance prediction [23] and explainable membrane-learning reviews [24] offer related but target-specific evidence. Such work establishes the feasibility of data-driven prediction but does not, by itself, validate transfer to unseen operating periods or plants.

Ensemble approaches have also been investigated for RO membrane-flux prediction [25], explainable osmotically assisted RO models [26], and plant permeate-flow modelling [27]. Other full-scale studies combine historical data with physical fouling descriptions [28], while a separate membrane-bioreactor study explicitly quantified model uncertainty [29]. Because these investigations use different membrane configurations, outputs, and validation designs, their numerical scores should not be compared directly with those from the present plant. These studies instead inform feature selection, robustness testing, and the need to report prediction uncertainty.

The present study therefore compares a regularized linear reference (Ridge) with three nonlinear regression ensembles (Random Forest, Extra Trees, and Gradient Boosting), selected to contrast different model assumptions rather than to rank algorithms universally [30,31,32]. Although Random Forest can model nonlinear operating relationships, its apparent retrospective performance may be inflated when temporally related records are divided randomly. Time-aware estimation studies document the importance of evaluation methods for nonstationary time series and structured observations [33,34,35,36]. Leakage terminology must also be applied carefully: having later-dated training rows in a random split makes that experiment non-prospective, even where held-out rows are not used during fitting [37].

Drift-aware model assessment distinguishes shifts in observed input distributions from an unverified change in the conditional relationship between inputs and outputs [38,39]. The analysis therefore describes the observed operating-regime and data shifts without attributing a unique physical root cause or claiming to have conclusively identified the concept drift. It treats random splitting as an interpolation benchmark and chronological, expanding-window, and later-period tests as progressively more deployment-relevant checks.

An earlier four-year investigation of the same facility examined operational stability, pretreatment, fouling indicators, membrane condition, and cleaning responses during 2020–2024 [40]. Its purpose was to characterize the physical process. In contrast, this paper uses a model-ready subset from June 2022 to November 2023 and an April–May 2024 holdout to examine the temporal validity of supervised predictions and the implications for a future advisory digital twin; it does not repeat the earlier Operational Stability Index or membrane-autopsy assessment. An offline RO digital-twin-assisted study similarly reported that certain quality outputs remained difficult to predict without sufficient membrane and feed characterization [41].

This study had six objectives: (i) to audit the variation in the structures of historical operational workbooks; (ii) to harmonize train-day records using explicit quality rules; (iii) to compare four supervised regression methods for second-stage permeate flow and conductivity; (iv) to evaluate random, chronological, rolling-origin, and later-period validation; (v) to examine distributional changes using standardized mean differences and Kolmogorov–Smirnov distances; and (vi) to derive the requirements for an RO digital-twin readiness framework. The analysis concerned retrospective and advisory model readiness, not a deployed, closed-loop, physics-coupled digital twin.

## 2. Materials and Methods

### 2.1. RO Process Context and Source Workbooks

The case study examined a full-scale drinking-water plant combining pre-treatment, microfiltration, and two-stage reverse osmosis. Operational measurements were recorded by treatment train and operating date, rather than obtained as a synchronized, high-frequency historian series. The available fields included microfiltration differential pressure, feed pressure, membrane-stage conductivity and flow, temperature, turbidity, and the silt density index (SDI). Some records also contained maintenance-related text, including cartridge replacement and cleaning-in-place (CIP) notes. These contextual entries were not sufficiently standardized for inclusion as common numerical predictors. Because this was a retrospective data-analysis study based on existing operational records, no additional laboratory equipment, materials, or devices were introduced specifically for the present analysis.

Two operational Microsoft Excel workbooks were assessed. The legacy weekly and monthly reporting archive contained 70 worksheets, including 58 with month labels; the month-labelled worksheets had 21 distinct column widths and several layouts. The second, more consistently structured workbook contained monthly train-level records and served as the source of the model-ready dataset. The former was assessed for structural variability and ingestion risks; it was not treated as an independent source of additional modelling observations. Table 1 summarizes the roles and time periods of the two sources. The source-to-advisory workflow used in the study is shown in Figure 1.

### 2.2. Canonicalization and Data-Quality Rules

The normalized monthly worksheets were harmonized into dated train-level records using source-specific column mappings and identity checks. Only dates matching their source worksheet month/year and train identifiers 1, 2, or 3 were retained. Historical records came from 17 nonempty operating-month worksheets (June–December 2022 and February–November 2023); January 2023 contained no operational records. Two rows dated 2 and 3 May 2023 were excluded from the April 2023 worksheet (source rows 33–34). The sheet labelled March 2024 was excluded because its internal heading and numeric Excel dates identify March 2023; it was not re-dated or used as independent 2024 evidence. April and May 2024 provided 30 and 25 train-day records, respectively. The workbook-level extraction and sheet/row provenance are documented in the accompanying data-audit register.

Eight predictors were selected because their source tags could be mapped consistently for both the historical model period and the later April–May 2024 evaluation: train identifier, microfiltration differential pressure, first-stage feed pressure, first- and second-stage differential pressures, first-stage permeate flow, microfiltered-water conductivity, and first-stage permeate conductivity (Table 2). The two targets were second-stage permeate flow and conductivity. The April–May 2024 source lacks turbidity, silt density index (SDI), and temperature; detailed feed chemistry, recovery, membrane age, and synchronized intervention histories were not consistently available. Their exclusion was an availability decision, not evidence that these covariates are irrelevant. CIP and operator notes remain contextual records and were not included as predictors. RO studies have independently identified temperature, pressure, flow and membrane age as potentially informative predictors [19,21,22]; their exclusion here limits inference rather than demonstrating physical irrelevance.

Missing numerical values were preserved in source extracts and imputed only after each training split had been established. One SDI entry of 28 on 24 April 2023 (sheet 4.2023, row 25) was flagged as implausible for SDI descriptive analyses: SDI completeness thus equals 331/476 rather than 332/476, while the original cell remains unchanged. Source sheet 3.2024 carries March 2023 in its internal heading and dates and was excluded rather than assigned an unsupported 2024 timestamp. Each retained record contains the source worksheet and row location, date, and train; no duplicate date–train keys were found. These checks constitute retrospective quality controls, not an implemented online digital-twin data-quality service.

### 2.3. Data Completeness and Historical Operating Envelope

Completeness was calculated as the proportion of train-day records containing a numeric value for each monitored variable. Descriptive statistics for 2022–2023 were computed from available values without imputation. Medians and interquartile ranges (IQRs) were emphasized because the historical record contains atypical and low-flow operating states. These statistics describe the observed data envelope and are not interpreted as membrane design limits. Record-level completeness is summarized in Table 3, and the historical descriptive baseline is presented in Table 4.

### 2.4. Machine-Learning Models

Four supervised regression algorithms were compared for each target. Ridge Regression provided a regularized linear reference. Random Forest averaged predictions from bootstrap-based trees [30], Extra Trees used additional randomization in ensemble tree construction [31], and Gradient Boosting fitted successive trees to improve an additive predictive model [32]. These complementary modelling assumptions were selected to examine whether the conclusions about temporal generalization persisted across a linear benchmark and several nonlinear ensemble approaches. Related RO literature has evaluated alternative and ensemble models for membrane-performance and flux prediction [19,25]. The objective here was to assess the sensitivity of model evaluation to the validation protocol, not to claim exhaustive algorithm selection. Previous industrial RO work has used dynamic networks and operating-window sensitivity [20], while ensemble studies have evaluated membrane flux and operating-variable effects [25,26]. These are methodological comparisons, not directly comparable predictive benchmarks.

Missing numerical predictors were imputed using medians fitted on the training fold only, and train identity was one-hot encoded. Numeric variables were standardized for Ridge Regression (α = 1). Random Forest used 500 trees, maximum depth 12, minimum leaf size 3; Extra Trees used 500 trees, maximum depth 12, minimum leaf size 2; and Gradient Boosting used 250 estimators, maximum depth 3, learning rate 0.03, and Huber loss. The scikit-learn implementation was used for all four regressors and preprocessing [42]. The exact scikit-learn package version was not retained in the archived analysis environment; therefore, no unverifiable version number has been introduced. Reproducibility checks used random_state = 42 for the random split and stochastic estimators; records for chronological evaluation were ordered by date and then train. Models used the same predictor set and fixed hyperparameters across all evaluation protocols. We additionally examined Random Forest depths 4, 8, 12, and unrestricted, and minimum leaf size 6 at depth 12, to assess sensitivity to model complexity without tuning on the later holdout.

### 2.5. Time-Aware Validation Protocols

Three validation protocols were used independently for each measured target: (i) a retrospective random 80/20 split of the eligible historical observations (random_state = 42); (ii) a chronological 80/20 split with sorting by operating date, then train (training N = 271, test N = 68 for flow; training N = 299, test N = 75 for conductivity); and (iii) fitting on all target-eligible historical observations and testing only on April–May 2024 (41 flow and 46 conductivity measurements). All imputation and scaling parameters were fitted only on the relevant training observations. The last protocol is a later-period holdout from the same site, not an independent external-plant validation.

Random splitting is reported solely as an interpolation benchmark. The split-specific median imputer was fitted only on random training rows; however, some such rows occurred after the dates of held-out test rows, so random-split preprocessing and model fitting are not temporally prospective. Chronological, rolling-origin, and 2024 holdout pipelines use only earlier-period observations for fitting all preprocessing and model parameters. Thus, the later-period deterioration is not produced by directly fitting imputers to the 2024 test data. Performance under a strictly prior-period training protocol is reported as the principal future-facing evaluation. This distinction is consistent with cautions about leakage and temporal dependence in broader predictive modelling [36,37].

For expanding-window rolling-origin validation, Random Forest was retrained using only observations preceding each of four future blocks: January–March, April–June, July–September, and October–November 2023. Each target used its own available-case training and test counts. R^2^, root mean square error (RMSE), and mean absolute error (MAE) were calculated on measured outcomes. As additional sensitivity analysis, fitted-model predictions were subjected to exploratory circular moving-block resampling: 3 consecutive test observations per bootstrap block and 3500 draws within each rolling test interval; and, for the April–May 2024 holdout, block length was 5 measured target observations and 4000 draws. Each draw resampled consecutive prediction–observation pairs while holding the trained model fixed; reported percentile limits are conditional uncertainty screens rather than formal prospective prediction intervals. Small, irregularly spaced and autocorrelated samples, plus omission of training-data uncertainty, limit their inferential interpretation. The preference for reporting multiple chronological assessment periods is consistent with time-series evaluation research [35].

### 2.6. Operating-Regime Drift Diagnostics

The historical 2022–2023 records were compared with the April–May 2024 holdout for variables measured in both periods. Standardized mean differences (SMDs) quantified mean shifts relative to pooled variation [43]; two-sample Kolmogorov–Smirnov distances (KS D) measured separation between empirical distributions [44]. KS *p*-values were reported as descriptive screening statistics because serial dependence and changes in sampling patterns may violate simple independence assumptions. Interpretation therefore combined medians, missingness, SMD, KS distance, and prediction errors rather than treating a small *p*-value as proof of a physical mechanism.

The term operating-regime drift is used as an engineering umbrella for observed distributional changes. Data drift refers specifically to changes in input distributions, while concept drift is reserved for changes in the input–output relationship. The available data demonstrate operating-regime and data drift directly; they do not, by themselves, prove a specific physical cause or a fully identified concept-drift mechanism. Published drift taxonomies likewise distinguish detectable distributional change from a demonstrated change in the input–output concept [38,39].

### 2.7. Digital-Twin Readiness Criteria

Model readiness was assessed against a staged architecture separating the physical RO plant, secure connectivity, a canonical information layer, model services, model-health monitoring, and operator-facing decision support. This conceptual decomposition is compatible with published digital-twin framework and information-exchange principles [45,46]. Proposed operational technology connectivity should incorporate access control and auditability [47]. The case study demonstrates a retrospective data and validation workflow; the architecture represents proposed capabilities, not functions already deployed at the plant. An advisory model should be withheld from decisions when data-quality criteria fail or operation leaves its validated envelope. Autonomous closed-loop optimization was excluded from scope.

## 3. Results

### 3.1. Data Readiness and Historical Membrane Operating Envelope

The legacy archive provides direct evidence of an evolving operational information environment. Among the 58 month-labelled worksheets, 21 distinct worksheet widths were observed. This is not merely a formatting issue: a positional ingestion rule could silently map different columns to the same process variable when the spreadsheet structure changes. Canonical tag mapping is therefore a prerequisite for trustworthy reuse of the historical record.

After the time/date and train-consistency checks, 476 historical train-day records covered 473 distinct calendar dates from 1 June 2022 to 30 November 2023. The difference is fully accounted for by three dates on which two different trains were recorded: 20 March 2023 (trains 2 and 1), 22 March 2023 (trains 1 and 3), and 27 March 2023 (trains 2 and 1). The pairs occur at worksheet rows 21/34, 23/35, and 28/36, respectively, in 3.2023. No (date, train) pair occurs twice. These are legitimate distinct train-day observations, not three duplicated records. Completeness ranged from 68.1% for microfiltered-water conductivity to 84.0% for stage-1 conductivity; second-stage flow and conductivity were present in 71.2% and 78.6% of historical records. The 55 later records contain no turbidity, SDI, or temperature readings.

The historical membrane dataset covered a broad range of operating states. The median stage-2 permeate flow was 93.6 m^3^/h and the median stage-2 permeate conductivity was 359 µS/cm. The median feed pressure was 12.30 bar. These values are used only to characterize the observed modelling envelope and to support later drift interpretation.

### 3.2. Apparent Predictive Performance Under Random Splitting

With an 80/20 random split, the four models showed a predictive signal for second-stage permeate flow, but performance differed by model and target (Table 5 and Table 6). Random Forest achieved R^2^ = 0.876, RMSE = 8.48 m^3^/h, and MAE = 4.68 m^3^/h for flow; and the Ridge baseline achieved R^2^ = 0.864. For conductivity, Extra Trees achieved R^2^ = 0.838, while Random Forest achieved R^2^ = 0.833, RMSE = 63.26 µS/cm, and MAE = 45.34 µS/cm. These values describe the predictive performance when training and testing the sample in the mixed historical period. They are informative as retrospective benchmarks, but do not measure accuracy under a later or previously unobserved operating regime.

### 3.3. Chronological and Later-Period Validation

Preserving the date order changed the evaluation (Table 5 and Table 6). For second-stage flow, Random Forest R^2^ declined from 0.876 with random splitting to 0.042 with chronological splitting, while its RMSE was 8.48 and 8.19 m^3^/h, respectively. For conductivity, its R^2^ declined from 0.833 to 0.118, with RMSE increasing from 63.26 to 86.42 µS/cm. Differences between R^2^ across test windows must also be interpreted in light of their different target variances; absolute-error metrics provide essential complementary information. Under chronological validation, Extra Trees yielded R^2^ = 0.189 for flow, whereas Gradient Boosting yielded R^2^ = 0.134 for conductivity. These results show that the gap was not restricted to one target or one algorithm.

The April–May 2024 holdout further challenged generalization after models were refitted on all eligible 2022–2023 observations. Random Forest achieved R^2^ = −1.173 for flow (RMSE = 11.14 m^3^/h; MAE = 9.15 m^3^/h) and R^2^ = −0.918 for conductivity (RMSE = 218.65 µS/cm; MAE = 173.66 µS/cm). The respective test sets contained only 41 and 46 complete target observations. Across the compared methods, Gradient Boosting yielded flow R^2^ = −0.534, while Ridge yielded conductivity R^2^ = −0.010; neither result establishes a reliable prediction of both outcomes. Negative R^2^ values indicate performance below the corresponding test-set mean baseline, but the magnitude and precision of the estimates require caution for this short, non-annual holdout. The validation-design effect is illustrated in Figure 2.

### 3.4. Rolling-Origin Robustness Check

The original data and deterministic re-execution reproduced all 24 main model-comparison results (Table 5 and Table 6) to the reported precision. For rolling-origin validation, the training/test sample counts matched the original report, but minor numerical differences remained; Table 7 has therefore been updated to the reproducible random_state = 42 outputs. Flow R^2^ was 0.685, 0.054, −0.975, and 0.474 across the four successive blocks. Conductivity R^2^ was −0.007, −0.210, −5.578, and 0.463. Exploratory 95% block-bootstrap percentile limits for the July–September block were [−2.200, −0.515] for flow and [−19.145, −2.520] for conductivity (Figure 3). The very wide conductivity range reflects the sensitivity of R^2^ to the test-set variance and the limited number of temporally dependent records. This indicates variable future-facing performance; it does not identify a particular fouling or process-change mechanism.

Additional robustness checks indicate that simplification alone did not eliminate the 2024 degradation. Random Forest flow, at maximum depth 4, achieved training/chronological-test R^2^ values of 0.898/−0.022 and full-historical-training/2024-test values of 0.892/−0.929; the original depth-12 setting gave 0.917/0.042 and 0.920/−1.173, respectively. Increasing the minimum leaf size from 3 to 6 at depth 12 improved the flow holdout R^2^ to −0.844, but did not make it positive. For conductivity, depth 4 produced training/2024-test R^2^ values of 0.867/−1.653; depth 12 gave 0.948/−0.918. Both high training scores and sensitivity to complexity warrant caution about overfitting, while negative later-period scores persist under simpler settings. These tests distinguish the robustness of the empirical warning from a causal proof of drift.

For the fixed depth-12 Random Forest on April–May 2024, the exploratory 95% moving-block percentile intervals for R^2^ were [−4.265, −0.387] for flow (41 observations) and [−2.286, −0.409] for conductivity (46 observations). The corresponding RMSE intervals were 8.73–12.99 m^3^/h and 139.96–281.72 µS/cm; the MAE intervals were 6.55–11.27 m^3^/h and 109.25–244.37 µS/cm. These conditional resampling screens indicate substantial numerical uncertainty in the magnitude of model deterioration; they do not establish population-wide coverage or rule out omitted-variable and measurement effects.

### 3.5. Operating-Regime Drift Between 2022–2023 and 2024

Relative to the historical dataset, the 2024 holdout exhibited a lower median feed pressure (12.30 versus 11.40 bar; SMD = −1.90; KS D = 0.766), higher first-stage permeate conductivity (74.0 versus 122.4 µS/cm; SMD = 1.08; KS D = 0.632), higher second-stage flow (93.6 versus 103.68 m^3^/h; SMD = 1.14; KS D = 0.547), and higher second-stage conductivity (359 versus 515.5 µS/cm; SMD = 0.88; KS D = 0.437). In contrast, second-stage differential pressure showed a smaller standardized mean difference of 0.36. These observations establish differences between the recorded distributions but do not identify whether the changes originated in water chemistry, membrane condition, control setpoints, or instrumentation. These standardized shifts are summarized in Figure 4, and the corresponding distribution-shift diagnostics are reported in Table 8.

### 3.6. Digital-Twin Readiness Outcome

The combined validation evidence supports a digital-shadow or advisory-twin pathway, but not immediate closed-loop optimization. The positive result is that legacy RO data can be transformed into a traceable canonical layer and can support useful interpolation models within familiar operating regimes. The key constraint is temporal stability: a model that performs well on randomized historical observations can become unreliable in a later membrane operating regime.

Accordingly, the target architecture stores the time series together with the train/asset identity, units, quality flags, schema version, and maintenance/CIP context. The model layer includes soft sensors, uncertainty or residual monitoring, drift detection, and candidate versions for retraining. Only approved versions should be exposed to applications, while measured and inferred values remain distinguishable so that the system can fall back to raw monitoring when the predictive layer leaves its validated envelope. The proposed staged architecture is shown in Figure 5, and the minimum readiness capabilities are summarized in Table 9.

## 4. Discussion

### 4.1. Temporal Generalization Gap Under Random and Chronological Validation

Random-split R^2^ exceeded 0.83 for both Random Forest targets, but the corresponding chronological values were only 0.042 for flow and 0.118 for conductivity. On the later 2024 holdout, both R^2^ values were negative. This contrast separates interpolation among mixed historical observations from prospective prediction under subsequent plant conditions. Because the test periods differed in target variance and size, the R^2^ gap alone cannot quantify a unique cause; the associated RMSE and MAE trends, rolling-origin results, and measured distribution shifts provide the broader interpretation.

The difference can arise without conventional test-row leakage. Random splitting distributes observations from similar operating periods across both subsets; it can also estimate training-only pre-processing quantities from observations occurring later than individual test records. In a future-facing deployment, such information is unavailable at prediction time. Chronological and rolling-origin evaluation more closely resemble sequential plant operation [33,34]. Nevertheless, the model complexity and omitted variables may also contribute to the observed degradation. The present results support a validation warning rather than a unique causal attribution. Related methodological research shows why blocked or repeated future-facing holdouts can differ from randomly shuffled evaluation under nonstationarity [35,36].

### 4.2. Process-Grounded Interpretation of the 2024 Drift

The April–May 2024 observations combined a lower feed pressure with a higher first-stage permeate conductivity and higher second-stage permeate flow and conductivity. In RO, the flow and solute transport are linked to pressure, feed composition, temperature, membrane permeability, recovery, and fouling or scaling phenomena [1,2]. However, these explanatory variables were not all recorded with sufficient completeness in the model-ready dataset. The observed shifts therefore identify a changed operating distribution, not a verified membrane deterioration or a specific physical mechanism. This interpretation is consistent with the importance of feed chemistry, temperature, and process-state variables reported in RO performance and fouling studies [3,21,28]. It does not identify which mechanism changed at the present facility.

Possible contributors include changed setpoints, feed-water chemistry, membrane or cartridge replacement, CIP events, train operation, and sensor calibration, but none can be confirmed from the reported records. Missing temperature, membrane age, recovery, and synchronized maintenance variables also permit unobserved confounding. Future data acquisition should align these variables with the train identity and timestamps before a model-health warning is interpreted as evidence of a particular plant intervention.

### 4.3. Relation to Previous Full-Scale RO Monitoring and Recent Membrane Digital-Twin Studies

The newly completed sensitivity analyses qualify competing explanations. Random Forest training scores exceeded chronological and later-period scores, which is consistent with some degree of model overfitting or train–test mismatch. However, shallower trees and larger minimum leaves did not restore positive performance on the 2024 holdout for both targets, so an overly deep model cannot be the sole practical explanation for the observed poor transfer. Large shifts in measured predictors and targets are directly documented, but the analysis does not isolate their physical origin or establish a change in the conditional input–output relationship. Resampling intervals on the 41- and 46-observation holdouts are wide and conditional on fixed models; additional independent periods, process-state covariates, and prospective validation are required. An uncertainty study on an MBR plant [29] supports reporting the model and prediction variability, but that study is a different membrane process and cannot calibrate intervals for the present RO plant.

Published RO case studies provide more specific comparisons than general AI reviews. Mohammed and colleagues modelled RO salt rejection using time-dependent and water-quality predictors [19], Dologlu and Sildir used four years of industrial process measurements for future permeate-flow modelling [20], and Odabaşı and colleagues compared learning methods and process variables for RO performance [21]. Kherraf and colleagues studied permeate conductivity using measured physicochemical variables [22], and Teng and Ng linked a fouling adsorption model to a data-driven model [28]. Talhami and colleagues examined membrane flux using experimental data compiled from multiple publications [25], while Chae and colleagues studied osmotically assisted RO, a distinct process [26]. None constitutes a matched evaluation on the present plant, targets, dates, feature set, and train composition, so a cross-paper ranking by R^2^ would be inappropriate.

The methodological distinction is more informative than a numerical comparison. Some reported RO studies explicitly included membrane age, temperature, chloride, or feed flow [19,21,22], whereas those covariates were missing or insufficiently aligned across both periods here. Work on membrane-model uncertainty [29] and nonstationary drinking-water prediction [14] further illustrates why retrospective fit alone does not establish operational reliability. Recent membrane-learning and fouling reviews [4,17,18] discuss the limits of generalizing from incomplete or scale-specific data. Accordingly, the observed drop in this study should be understood as a site-specific validation result, not proof that any machine-learning family is intrinsically unsuitable for RO.

### 4.4. Requirements for a Credible RO Digital Twin

An RO digital-twin pathway should use a controlled tag dictionary, train and asset identifiers, synchronized timestamps, recorded units, explicit missingness and quality flags, and a preserved source audit. Model governance should record training windows, target definitions, feature lists, performance by validation period, drift-monitoring criteria, version ownership, and rollback rules. Maintenance and CIP events should be time-stamped and connected to the affected train. These practices extend the conceptual digital-twin information exchange [45,46] and OT governance expectations [47] to the data problems observed here. Model predictions should remain labelled as inferred values, separate from sensor measurements, and should be withheld from operational recommendations outside a validated envelope.

### 4.5. Limitations and Future Work

The evidence derives from one full-scale facility, daily train-level workbooks rather than synchronized historian measurements, and a two-month holdout (41 flow and 46 conductivity measurements). The temperature, turbidity, and SDI are wholly missing in that later period, and the feed chemistry, membrane age, recovery, energy, and aligned CIP/maintenance data are incomplete. The holdout trains are T1 and T2, whereas historical data also include T3; model transfer therefore combines temporal, operating-state, and train-composition changes. Exploratory moving-block intervals are sensitive to block selection, irregular observation spacing, and fixed-model conditioning; neither they nor the KS test *p*-values supply a causal diagnosis.

The workbook audit reconciled 476 historical train-day observations with 473 dates: three dates have records for two distinct trains, with no repeated date–train key. An empty January 2023 sheet, two date-mismatched rows in an April 2023 sheet, and the mislabelled March 2024 worksheet were handled under traceable inclusion rules. The recomputed principal model scores matched the originally reported precision, whereas Table 7 and Figure 3 were updated to reproducible fixed-seed values. Sensitivity and conditional resampling checks were added, but cannot establish the reason for drift or substitute for prospective validation. A longer independent follow-up with aligned feed chemistry, membrane age, operating state, and CIP events remains necessary.

## 5. Conclusions

The study evaluated the predictive-model readiness for an envisaged RO digital-twin pathway rather than implementing a live twin. Seventy legacy worksheets showed heterogeneous structures; a normalized source yielded 476 historical train-day records over 473 distinct dates from 17 operating months, plus 55 later records from April to May 2024. Three March 2023 dates legitimately contained measurements from two different trains, while two date-mismatched rows and one mislabeled March 2024 worksheet were excluded according to the stated provenance rules.

All 24 principal model-comparison results were reproduced from the source workbooks to the reported precision. The Random Forest random-split R^2^ was 0.876 for stage-2 flow and 0.833 for conductivity (RMSE 8.48 m^3^/h and 63.26 µS/cm); the chronological values were 0.042 and 0.118, and later-period values −1.173 and −0.918 (RMSE 11.14 m^3^/h and 218.65 µS/cm). The rolling-origin results, recalculated with an explicit seed, differed modestly from the prior summary but remained unstable. Even shallower forests yielded a negative later-period R^2^, while exploratory conditional 95% block-bootstrap percentile intervals for depth-12 R^2^ were [−4.265, −0.387] for flow and [−2.286, −0.409] for conductivity. The measured operating-state differences coexist with prediction failures; neither overfitting nor a unique physical drift mechanism can be fully isolated.

The practical implication is to require stable data contracts, verified source provenance, temporally separated validation, maintenance context, model monitoring, controlled retraining, and operator-supervised advisory operation. The results do not validate automated process control or resource-efficiency gains. The evidence remains limited by a single plant, an incomplete common feature set, short later-period evaluation, and exploratory conditional uncertainty estimates; longer prospective assessments are needed prior to operational deployment.

## Figures and Tables

**Figure 1:**
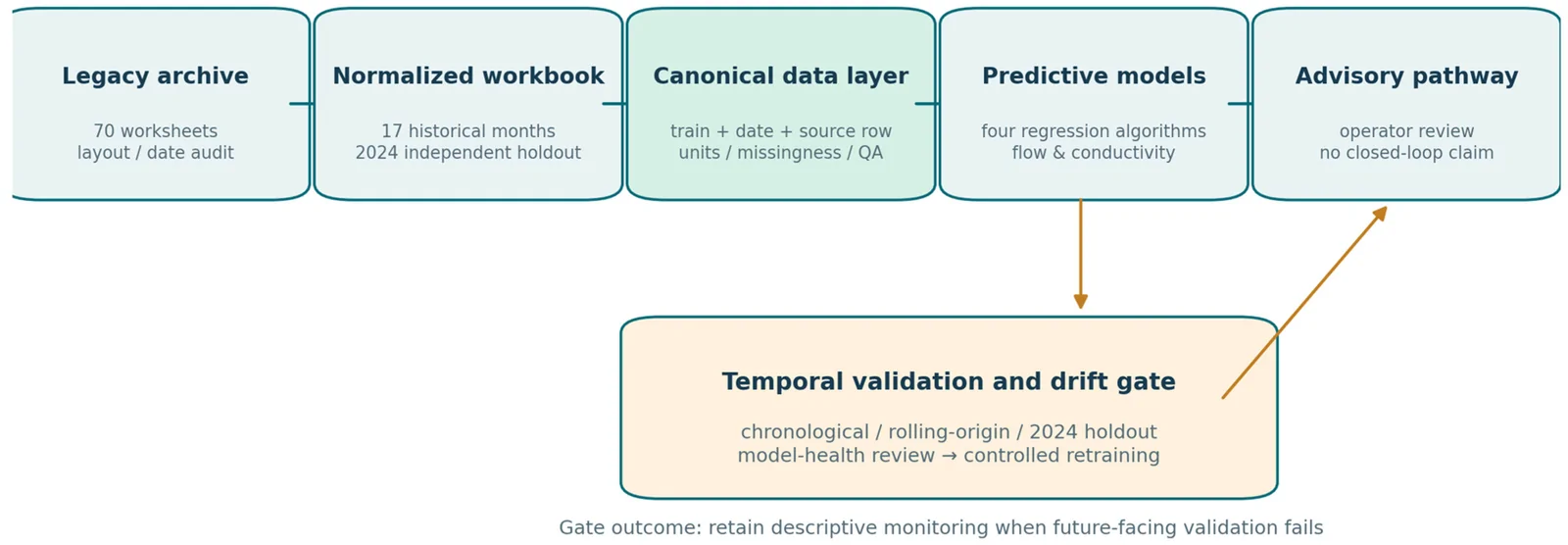
Source-to-advisory workflow illustrating the proposed RO data foundation and validation gate. The historical archive is used for schema auditing, the normalized workbook for modelling; models are not deployed when future-facing validation fails. This workflow is conceptual and does not imply an implemented online system.

**Figure 2:**
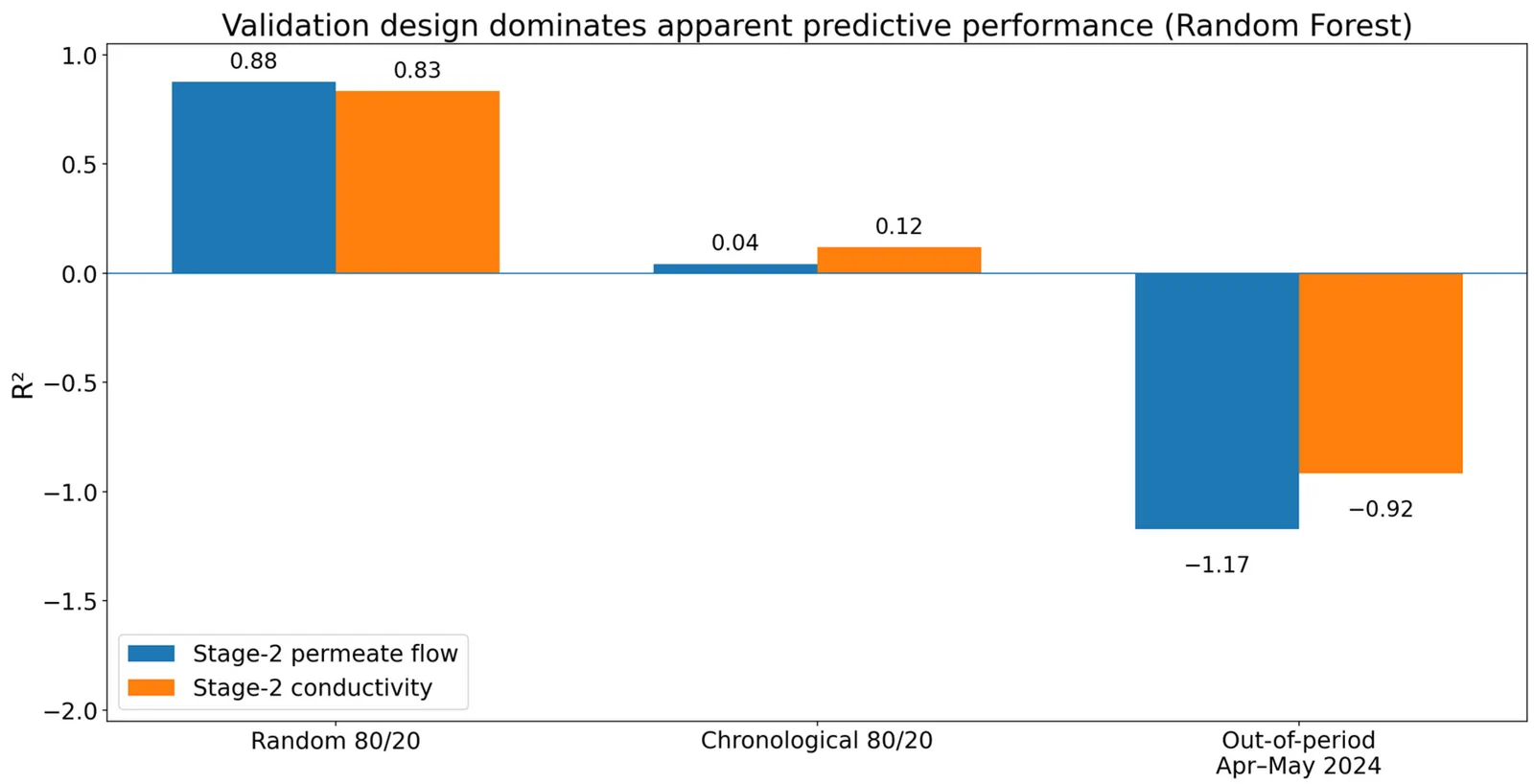
Random Forest R^2^ under random, chronological, and later-period validation. The validation gap shows that random splitting substantially overstates future-facing generalization for both RO targets.

**Figure 3:**
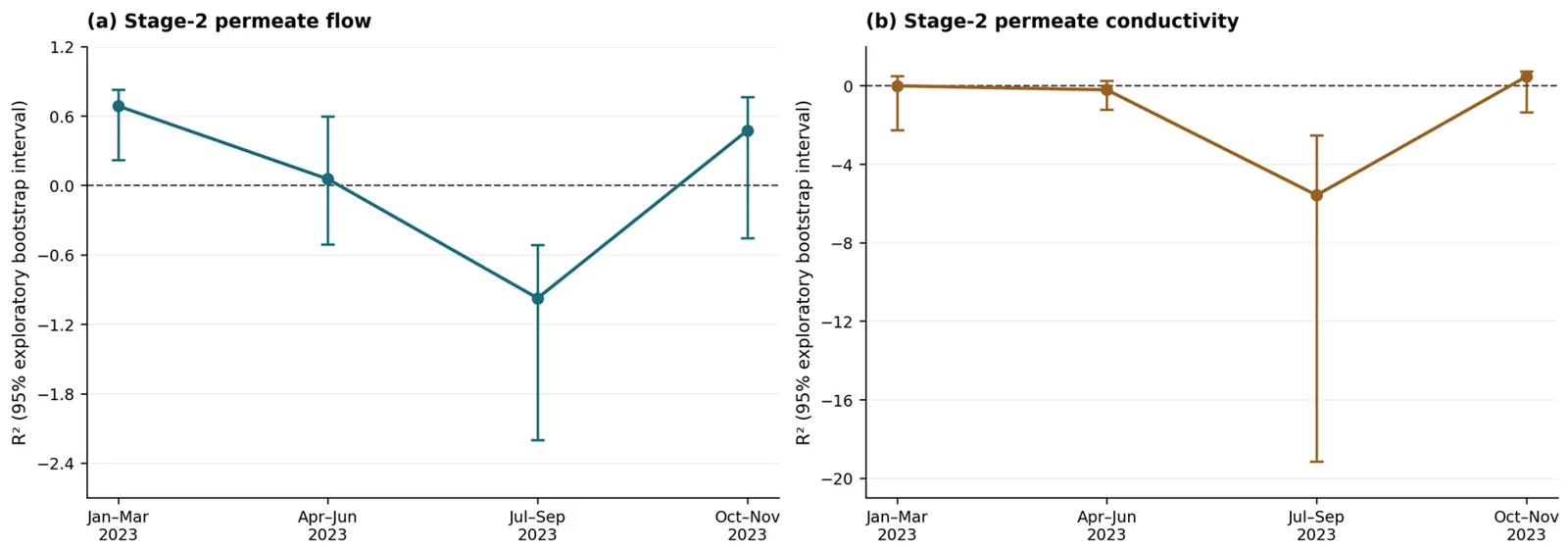
Rolling-origin Random Forest R^2^ for (**a**) second-stage permeate flow and (**b**) conductivity. Points are recomputed performance estimates; whiskers give exploratory 2.5th–97.5th percentile intervals from 3500 circular moving-block bootstrap draws of three consecutive target-available observations. The fitted model is fixed; intervals do not account for training uncertainty or irregular sampling and should not be interpreted as calibrated prospective confidence bands. No embedded figure title is used.

**Figure 4:**
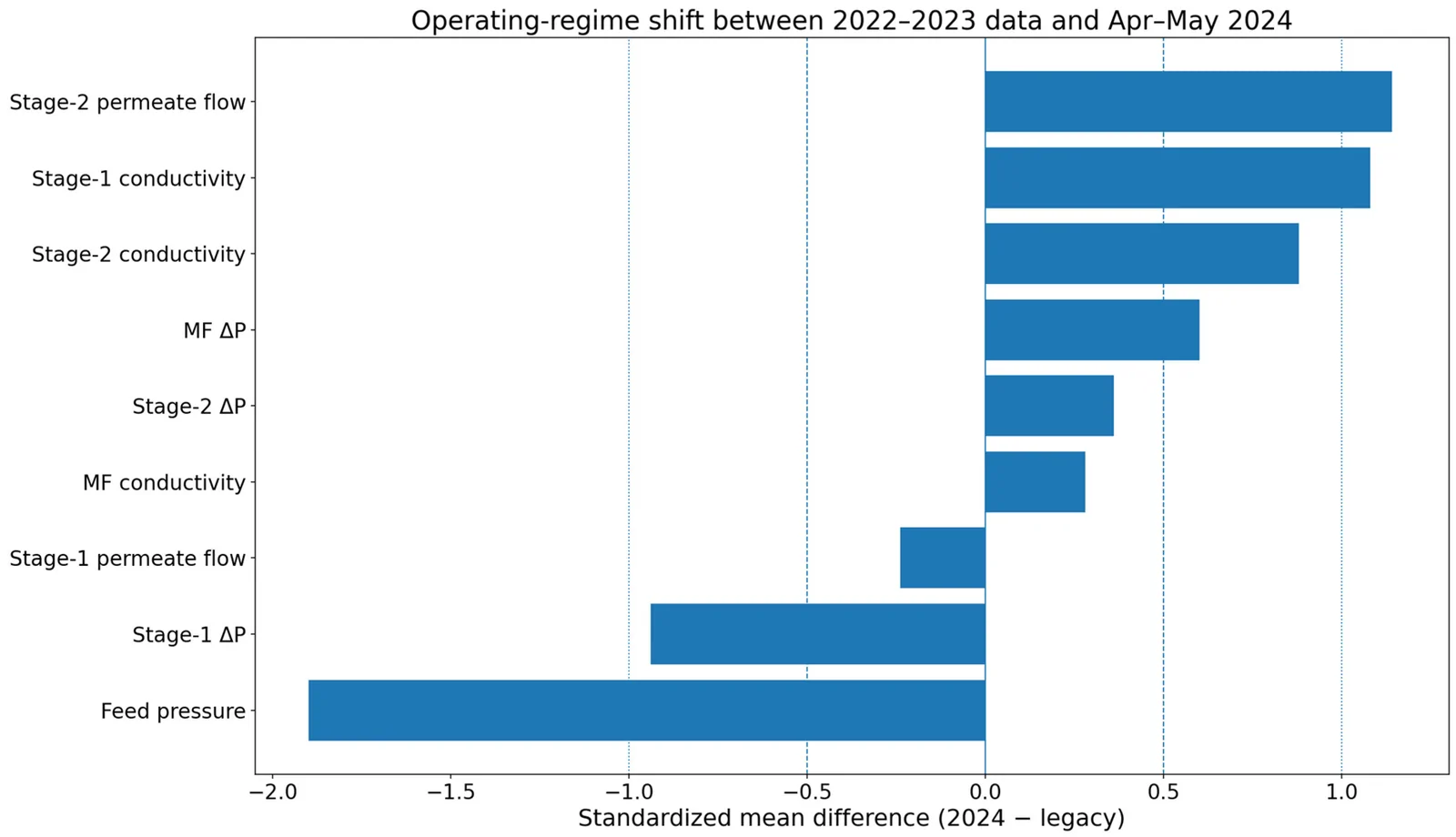
Standardized mean differences between the historical modelling distribution and the April–May 2024 holdout. Magnitudes above 1 indicate large shifts relative to pooled variability.

**Figure 5:**
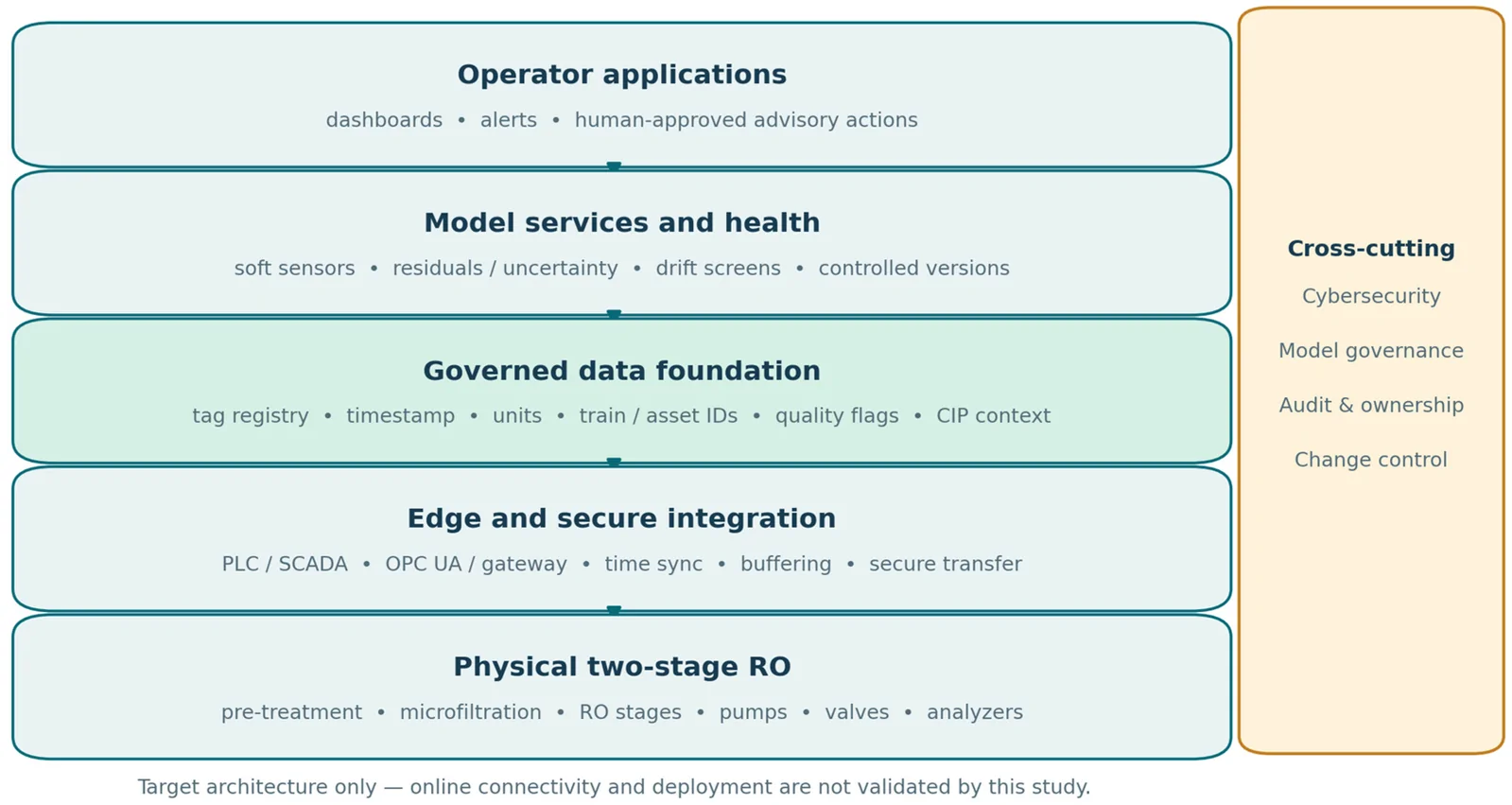
Proposed staged RO digital-twin architecture, with governance and cybersecurity functions extending across physical equipment, connectivity, data, models and operator applications. The diagram depicts a target design, not operational deployment or validated closed-loop control.

**Table 1:** Data sources, study periods, and inclusion logic.

Data Source/Period	Observed Structure	Use in Study	Decision
Legacy reporting archive	70 worksheets; 58 month-labelled sheets	Structural audit	Evidence of schema evolution and variant layouts
Normalized operational workbook	Monthly train-level worksheets	Model development/validation	Primary canonical source
Legacy modelling period	June 2022–November 2023	476 train-day rows	17 operating-month sheets after checks
Later-period holdout	April–May 2024	55 train-day rows	Reserved for out-of-period validation
Excluded March 2024 sheet	Title says 2024; internal dates say March 2023	Excluded	Excluded under the documented month/date-consistency rule

**Table 2:** Canonical variables retained for the common time-aware model comparison.

Canonical Tag	Process Meaning	Unit	Role
train	RO train identifier	categorical	Predictor
dp_mf_bar	Microfilter differential pressure	bar	Predictor
p_feed1	Stage-1 feed pressure	bar	Predictor
dp_e1	Stage-1 differential pressure	bar	Predictor
dp_e2	Stage-2 differential pressure	bar	Predictor
q_p1	Stage-1 permeate flow	m^3^/h	Predictor
cond_mf	Microfiltered-water conductivity	µS/cm	Predictor
cond_p1	Stage-1 permeate conductivity	µS/cm	Predictor
q_p2	Stage-2 permeate flow	m^3^/h	Target
cond_p2	Stage-2 permeate conductivity	µS/cm	Target

**Table 3:** Record-level completeness in the 2022–2023 modelling period and April–May 2024 holdout.

Variable	Legacy N	Legacy Complete	2024 N	2024 Complete
Train ID	476	100.0%	55	100.0%
Turbidity	400	84.0%	0	0.0%
SDI	331	69.5%	0	0.0%
Temperature	389	81.7%	0	0.0%
MF ΔP	337	70.8%	42	76.4%
MF conductivity	324	68.1%	54	98.2%
Stage-1 conductivity	400	84.0%	46	83.6%
Stage-2 conductivity	374	78.6%	46	83.6%
Stage-1 permeate flow	349	73.3%	41	74.5%
Stage-2 permeate flow	339	71.2%	41	74.5%
Feed pressure	358	75.2%	41	74.5%
Stage-1 ΔP	358	75.2%	41	74.5%
Stage-2 ΔP	344	72.3%	41	74.5%

**Table 4:** Descriptive baseline for the 2022–2023 harmonized dataset. Statistics use available values; missing values are not imputed for this table.

Variable	N	Mean	Median	IQR	Range
Turbidity (NTU)	400	0.13	0.12	0.10–0.14	0.07–0.90
SDI	331	1.96	2.08	1.61–2.30	0.11–4.25
Temperature (°C)	389	18.87	19.50	17.20–21.10	10.00–28.80
MF ΔP (bar)	337	0.43	0.39	0.31–0.48	0.12–1.11
MF conductivity (µS/cm)	324	2832.85	2820.00	2760.00–2890.00	2220.00–3210.00
Stage-1 permeate conductivity (µS/cm)	400	90.16	74.00	65.00–122.40	48.00–198.40
Stage-2 permeate conductivity (µS/cm)	374	407.43	359.00	281.25–557.00	69.70–848.00
Stage-1 permeate flow (m^3^/h)	349	276.02	277.20	273.60–280.80	77.00–295.20
Stage-2 permeate flow (m^3^/h)	339	81.94	93.60	50.40–98.80	10.70–133.60
Feed pressure (bar)	358	12.27	12.30	11.90–12.68	10.70–13.50
Stage-1 ΔP (bar)	358	2.91	2.70	2.50–3.00	1.60–7.70
Stage-2 ΔP (bar)	344	2.82	2.90	2.70–3.00	1.60–3.40

**Table 5:** Comparison of models predicting second-stage permeate flow. Train/Test N reports eligible observations in each split. Random and chronological training used 80% subsets of June 2022–November 2023; later-period evaluation trained on all eligible June 2022–November 2023 observations. RMSE and MAE are in m^3^/h.

Model	Validation	Train/Test N	R^2^	RMSE	MAE
Ridge	Random 80/20	271/68	0.864	8.91	5.29
Ridge	Chronological 80/20	271/68	−0.673	10.82	8.03
Ridge	Out-of-period Apr–May 2024	339/41	−1.886	12.83	11.46
Random Forest	Random 80/20	271/68	0.876	8.48	4.68
Random Forest	Chronological 80/20	271/68	0.042	8.19	5.48
Random Forest	Out-of-period Apr–May 2024	339/41	−1.173	11.14	9.15
Extra Trees	Random 80/20	271/68	0.868	8.75	4.60
Extra Trees	Chronological 80/20	271/68	0.189	7.54	5.22
Extra Trees	Out-of-period Apr–May 2024	339/41	−1.261	11.36	9.83
Gradient Boosting	Random 80/20	271/68	0.857	9.12	4.87
Gradient Boosting	Chronological 80/20	271/68	−0.084	8.71	6.01
Gradient Boosting	Out-of-period Apr–May 2024	339/41	−0.534	9.36	7.02

**Table 6:** Comparison of models predicting second-stage permeate conductivity. Train/Test N reports eligible observations in each split. Random and chronological training used 80% subsets of June 2022–November 2023; later-period evaluation trained on all eligible June 2022–November 2023 observations. RMSE and MAE are in µS/cm.

Model	Validation	Train/Test N	R^2^	RMSE	MAE
Ridge	Random 80/20	299/75	0.712	83.09	63.76
Ridge	Chronological 80/20	299/75	−0.886	126.37	104.60
Ridge	Out-of-period Apr–May 2024	374/46	−0.010	158.69	125.23
Random Forest	Random 80/20	299/75	0.833	63.26	45.34
Random Forest	Chronological 80/20	299/75	0.118	86.42	65.52
Random Forest	Out-of-period Apr–May 2024	374/46	−0.918	218.65	173.66
Extra Trees	Random 80/20	299/75	0.838	62.33	44.46
Extra Trees	Chronological 80/20	299/75	−0.149	98.61	84.63
Extra Trees	Out-of-period Apr–May 2024	374/46	−1.052	226.13	175.21
Gradient Boosting	Random 80/20	299/75	0.800	69.36	49.93
Gradient Boosting	Chronological 80/20	299/75	0.134	85.63	68.27
Gradient Boosting	Out-of-period Apr–May 2024	374/46	−0.909	218.14	174.56

**Table 7:** Recomputed Random Forest expanding-window rolling-origin performance (random_state = 42). Train N and test N refer to observations with the measured target; flow errors are in m^3^/h and conductivity errors are in µS/cm. Exploratory per-block R^2^ uncertainty is illustrated in Figure 3; numerical intervals and resampling assumptions are supplied in the audit workbook.

Future Block	Flow Train N	Flow Test N	Flow R^2^	Flow RMSE	Flow MAE	Cond. Train N	Cond. Test N	Cond. R^2^	Cond. RMSE	Cond. MAE
Jan–Mar 2023	181	38	0.685	13.63	10.18	189	17	−0.007	71.70	62.48
Apr–Jun 2023	219	55	0.054	20.48	9.28	206	78	−0.210	194.78	144.73
Jul–Sep 2023	274	27	−0.975	6.02	4.64	284	47	−5.578	114.55	95.53
Oct–Nov 2023	301	38	0.474	6.05	4.75	331	43	0.463	74.43	56.36

**Table 8:** Descriptive distribution-shift diagnostics comparing June 2022–November 2023 with April–May 2024. SMD is the standardized mean difference (2024 minus historical); KS D and KS *p* are the Kolmogorov–Smirnov distance and associated screening *p*-value.

Variable	Legacy Median	2024 Median	SMD	KS D	KS *p*
MF ΔP	0.39	0.56	0.60	0.388	1.35 × 10^−5^
Feed pressure	12.30	11.40	−1.90	0.766	1.34 × 10^−22^
Stage-1 ΔP	2.70	2.30	−0.94	0.535	2.65 × 10^−10^
Stage-2 ΔP	2.90	2.90	0.36	0.165	2.43 × 10^−1^
Stage-1 permeate flow	277.20	272.88	−0.24	0.341	2.69 × 10^−4^
Stage-2 permeate flow	93.60	103.68	1.14	0.547	9.86 × 10^−11^
MF conductivity	2820.00	2905.00	0.28	0.401	3.62 × 10^−7^
Stage-1 conductivity	74.00	122.40	1.08	0.632	2.48 × 10^−16^
Stage-2 conductivity	359.00	515.50	0.88	0.437	1.25 × 10^−7^

**Table 9:** Minimum capabilities recommended before advancing from spreadsheet-based RO analytics to an operational digital-twin service.

Capability	Minimum Implementation	Reason Supported by Case-Study Evidence
Canonical schema	Stable tag dictionary, units, train/asset IDs, timestamps, quality flags, schema versions	Prevents silent column/tag misalignment when structures evolve
Online data pipeline	PLC/SCADA or gateway connectivity, synchronized timestamps, buffering, historian	Replaces manual monthly ingestion with traceable time-series data
Context layer	CIP, cartridge changes, train state, membrane age, alarms, maintenance and operator actions	Separates process drift from maintenance/state transitions
Model validation	Random benchmark + chronological validation + later-period holdout	Tests interpolation and future-facing generalization
Drift monitoring	SMD/KS screening, residual trends, missingness and range checks	Triggers review before a stale model generates recommendations
Human-supervised service	Advisory output, explanation, operator acceptance/rejection log	Builds trust and prevents unvalidated automatic actions
Cybersecurity & governance	Least privilege, secure gateways, audit logs, model/version ownership	Protects OT and provides lifecycle accountability

## Data Availability

The original contributions presented in the study are included in the article. Further inquiries can be directed to the corresponding author.
